# Intravenous Nanoemulsions Loaded with Phospholipid Complex of a Novel Pyrazoloquinolinone Ligand for Enhanced Brain Delivery

**DOI:** 10.3390/pharmaceutics17020232

**Published:** 2025-02-11

**Authors:** Tijana Stanković, Tanja Ilić, Branka Divović Matović, Milos Petkovic, Vladimir Dobričić, Ivan Jančić, Biljana Bufan, Kristina Jezdić, Jelena Đoković, Ivana Pantelić, Danijela Randjelović, Dishary Sharmin, James M. Cook, Miroslav M. Savić, Snežana Savić

**Affiliations:** 1 Department of Pharmaceutical Technology and Cosmetology, Faculty of Pharmacy, University of Belgrade, 11211 Belgrade, Serbia; tijana.stankovic@pharmacy.bg.ac.rs (T.S.); jelena.djokovic@pharmacy.bg.ac.rs (J.Đ.); ivana.pantelic@pharmacy.bg.ac.rs (I.P.); snezana.savic@pharmacy.bg.ac.rs (S.S.); 2 Department of Pharmacology, Faculty of Pharmacy, University of Belgrade, 11211 Belgrade, Serbia; branka.divovic@pharmacy.bg.ac.rs (B.D.M.); kristina.mirkovic@pharmacy.bg.ac.rs (K.J.); miroslav.savic@pharmacy.bg.ac.rs (M.M.S.); 3 Department of Organic Chemistry, Faculty of Pharmacy, University of Belgrade, 11211 Belgrade, Serbia; milos.petkovic@pharmacy.bg.ac.rs; 4 Department of Pharmaceutical Chemistry, Faculty of Pharmacy, University of Belgrade, 11211 Belgrade, Serbia; vladimir.dobricic@pharmacy.bg.ac.rs; 5 Department of Microbiology and Immunology, Faculty of Pharmacy, University of Belgrade, 11211 Belgrade, Serbia; ivan.jancic@pharmacy.bg.ac.rs (I.J.); biljana.bufan@pharmacy.bg.ac.rs (B.B.); 6 Institute of Chemistry, Technology and Metallurgy, National Institute of the Republic of Serbia, University of Belgrade, 11000 Belgrade, Serbia; danijela@nanosys.ihtm.bg.ac.rs; 7 Department of Chemistry and Biochemistry, Milwaukee Institute for Drug Discovery, University of Wisconsin-Milwaukee, Milwaukee, WI 53211, USA; dsharmin@uwm.edu (D.S.); capncook@uwm.edu (J.M.C.)

**Keywords:** blood–brain barrier, droplet size, surface chemistry, neuropharmacokinetics, brain targeting

## Abstract

**Background/Objectives**: The novel pyrazoloquinolinone ligand CW-02-79 shows a unique profile of selective binding to σ2 receptors, but its poor solubility in both water and lipids makes its research and development a burdensome task. We aimed to develop a phospholipid-complex-based nanoemulsion formulation containing CW-02-79 suitable for intravenous administration in preclinical research. **Methods**: The decorated and undecorated nanoemulsions were formulated and subjected to detailed physiochemical characterization. The delivery and exposure to CW-02-79 from selected nanoemulsions were examined in the in vitro blood–brain barrier model based on human-induced pluripotent stem-cell-derived microvascular endothelial cells, astrocytes, and pericytes, and in vivo neuropharmacokinetic study in rats, respectively. **Results**: The developed biocompatible nanoemulsions loaded with a CW-02-79—phospholipid complex at a mass ratio of 1:10 exhibited a small droplet size and narrow size distribution, with satisfactory physicochemical stability during steam sterilization and short-term storage at 25 °C. The analysis of protein binding interactions revealed that the PEGylated nanoemulsions had fewer observable interactions compared to the undecorated nanoemulsions, especially when 0.2% DSPE-PEG2000 and 0.1% DSPE-PEG2000-mannose were combined. An in vitro BBB study demonstrated that a substantial part of CW-02-79 present in the applied nanoemulsion is able to permeate the barrier. The quantification of CW-02-79 in plasma/brain homogenate and calculated pharmacokinetic parameters confirmed good systemic and brain availability after intravenous administration. There were subtle differences in the pharmacokinetic parameters in favor of a dual surface-functionalized nanoemulson containing the glucose transporter-1-targeting ligand (mannose). **Conclusions**: The developed and characterized nanoemulsions enable substantial brain exposure to CW-02-79 as a prerequisite for a pharmacologically and clinically relevant selective modulation of σ2 receptors.

## 1. Introduction

Due to the substantial global burden and unmet needs of a variety of psychiatric and neurological disorders, tremendous efforts have been made to develop new chemical entities with novel or improved mechanisms of action. The pyrazoloquinolinone structure was recognized as the first non-benzodiazepine ligand to interact with the benzodiazepine site of GABA_A_ receptors [1] and has more recently yielded new ligands with functional selectivity for GABA_A_ receptors that contain the α6 subunit [2].

The primary screening of a novel pyrazoloquinolinone ligand CW-02-79 (7-methoxy-6-methyl-2-(4-(trifluoromethoxy)phenyl)-2,5-dihydro-3H-pyrazolo [4,3-c]quinolin-3-one) on 47 receptors and other targets within the Psychoactive Drug Screening Program (PDSP) discovered a total of five potential binding sites. Binding studies showed that CW-02-79 exerts at least a 20-fold selectivity for σ2 receptors. It has a substantial affinity for σ2 receptors (Ki value of 112 nM), with only low to negligible affinities at the other four hits discovered within the primary screen: 5-HT_7_ receptor (Ki = 2.4 µM), the benzodiazepine binding site at GABA_A_ receptors (Ki = 4.7 µM), histamine H_2_ receptor (Ki = 5.2 µM), and dopamine transporter (Ki > 10 µM) (available at: https://pdspdb.unc.edu/databases/kidb.php (accessed on 28 December 2024)). In vitro electrophysiological data demonstrated that CW-02-79 at a high concentration (10 µM) does not at all modulate GABA_A_ receptors that contain any of the α1 to α6 subunits (personal communication with Dr. Janet Fisher). Accordingly, the behavioral screening of this ligand in the wide range of doses (1 to 30 mg/kg) revealed no signs of sedation or motor incapacitation typical of commonly used benzodiazepines (unpublished data, MM Savić and B Divović Matović).

While σ2 receptors are mainly known for their high expression in rapidly proliferating cancer cells [3], they have increasingly been shown to play an important role in neuroprotection and neurological/cognitive disorders and are therefore potential targets for drug development for the treatment of brain diseases [4,5]. All these data point to CW-02-79 as a lead structure for the development of innovative psychopharmacological therapies that act via σ2 receptors. However, the undesirable physicochemical properties of CW-02-79, in particular its poor solubility in water and oils as well as in biocompatible organic solvents, jeopardize the development of a suitable parenteral formulation, which is required for extensive preclinical studies.

Phospholipid complex technology has been intensively investigated to address the poor solubility and permeability through the biological barriers of synthetic and naturally occurring active compounds, due to the high biocompatibility and biodegradability of the phospholipids. The numerous studies indicate that the formation of a phospholipid complex through the non-covalent interactions between drugs containing hydroxyl, carbonyl, and amine groups and the polar head of phospholipids can improve the lipophilicity of drugs and subsequently, their bioavailability [6,7,8]. However, due to stability issues (e.g., the risk of aggregation and chemical degradation) and formulation stickiness, drug-phospholipid complexes are commonly utilized as lipophilic intermediates for encapsulation into nanocarriers [9,10,11]. For example, the combinational approach based on drug–phospholipid complexes and intravenous nanoemulsions was shown to ensure an improved loading capacity for hydrophobic drugs without premature leakage, higher control over the drug release kinetics, and protection from undesirable hydrolytic and enzymatic degradation (e.g., [12,13,14]).

To achieve the efficient concentration of a drug in the brain after intravenous administration of nanoemulsions, multiple biological hurdles need to be overcome, including (i) opsonization and rapid clearance via the mononuclear phagocytic system (MPS) and (ii) the blood–brain barrier (BBB) [15,16]. The surface coating with PEGylated phospholipids is a commonly reported strategy to prolong the residence time of nanodroplets in the systemic circulation, thus increasing the likelihood for the brain’s uptake of nanodroplets [17,18]. Due to the limited specificity of this approach for brain delivery, an additional modification of the nanodroplet surface with a suitable ligand that targets the specific receptors on the brain endothelial cells is commonly required. In this study, mannose was used as a brain-targeting ligand due to its high affinity for glucose transporter-1 (GLUT-1) receptors, overexpressed on the BBB luminal side [19,20]. Mannose-coated nanoparticles have been reported to be able to enhance the drug delivery to the brain (e.g., by Lei et al. [21]). Therefore, to facilitate the interaction between the anchored mannose and GLUT-1 receptors, we used a relatively novel, commercially available excipient, DSPE-PEG2000-mannose, containing mannose attached at the terminus of the PEG chain as a spacer. Although this excipient was successfully employed on polymer nanoparticles [22] and liposomes [23], to the best of our knowledge, there are no reports on its use in the lipid nanoemulsions for brain targeting.

In this research, we aimed to investigate the potential of a two-fold formulation strategy based on a phospholipid complex and nanoemulsion to overcome the poor aqueous and oil solubility of CW-02-79, and thus to obtain a stable formulation suitable for intravenous administration. In addition, some of nanoemulsions were surface-functionalized/decorated with the PEGylated phospholipid (DSPE-PEG2000), alone or in combination with the GLUT-1 targeting ligand (DSPE-PEG2000-mannose), to enhance the brain targeting. After the high-pressure homogenization, nanoemulsions were characterized in terms of critical quality attributes and stability after the storage and steam sterilization. The permeation of CW-02-79 across the BBB from dual-modified/decorated and plain, undecorated nanoemulsions was assessed using a novel in vitro BBB model based on human induced pluripotent stem cell (iPSC)-derived cells. Finally, we evaluated the complete neuropharmacokinetic profiles of CW-02-79 after the single intravenous administration of the two most promising nanoemulsions to male rats, to elucidate the feasibility of this formulation type for preclinical studies.

## 2. Materials and Methods

### 2.1. Materials

CW-02-79 was synthesized at the Department of Chemistry and Biochemistry, University of Wisconsin-Milwaukee, USA. The following excipients were used for the nanoemulsion preparation: medium-chain triglycerides and castor oil were obtained from Fagron GmbH & KG, Glinde, Germany; soybean oil (Lipoid Purified Soybean Oil 700), soybean lecithin (Lipoid S75), and DSPE-PEG2000 (N-(Carbonyl-methoxypolyethylenglycol-2000)-1,2-distearoyl-sn-glycero-3 phosphoethanolamine, sodium salt) were purchased from Lipoid GmbH (Ludwigshafen, Germany); DSPE-PEG2000-Mannose (1,2-distearoyl-sn-glycero-3-phosphoethanolamine-N-(polyethyleneglycol)-Mannose, sodium salt) was provided by Biopharma PEG Scientific Inc., Watertown, MA, USA; glycerol, polysorbate 80, and butylhydroxy toluene (BHT) were supplied by Sigma-Aldrich Co. (St. Louis, MO, USA). Organic solvents (absolute ethanol, methanol, ethanol, dimethyl sulfoxide) were obtained from Fisher scientific (Waltham, MA, USA). Ultrapure water obtained by employing a GenPure apparatus (TKA Wasseranfbereitungssysteme GmbH, Neiderelbert, Germany) was used.

### 2.2. Preformulation Study

#### 2.2.1. Solubility Evaluation

The solubility of CW-02-79 in selected oils, organic solvents, and water solutions of various pH values was examined by using the shake-flask method, by adding an excess amount of CW-02-79. The blends were mixed on a vortex mixer for 24 h at 25 ± 2 °C to reach equilibrium. The samples were centrifuged (1000× *g*, 10 min, 25 °C) on a MiniSpin^®^ plus centrifuge (Eppendorf, Hamburg, Germany). The concentration of CW-02-79 was determined after proper dilution of the supernatant with isopropanol using the LC-MS/MS method.

#### 2.2.2. Polarization Microscopy

An initial insight into the physical state of CW-02-79 was obtained using polarized light microscopy. It was performed using an Olympus BX53-P microscope (Olympus, Tokyo, Japan) with cross polarizers. Photomicrographs were taken using the digital camera UPLFLN4XP and UPLFLN10XP, and processed with appropriate software, i.e., cellSens Entry Version 1.14 (Olympus, Tokyo, Japan).

#### 2.2.3. X-Ray Powder Diffraction Analysis (XRPD)

CW-02-79 was further analyzed on a Rigaku Smartlab X-ray Diffractometer in θ-θ geometry (the sample in the horizontal position) by applying parafocusing Bragg–Brentano geometry using a D/teX Ultra 250 strip detector in the 1D standard mode with a CuKα1,2 radiation source (U = 40 kV and I = 30 mA). The XRPD patterns were collected in the 3–40° 2θ range, with steps of 0.01° and a data collection speed of 3°/min. To minimize the background, the low-background single-crystal silicon sample holder was used.

#### 2.2.4. Melt-Quenching Approach

For this analysis, around 2 mg of the unprocessed CW-02-79 was heated to 330 °C, with a heating rate of 20 °C/min, then cooled to 25 °C with a cooling rate of 40 °C/min and finally heated again to 330 °C with a heating rate of 10 °C/min. The measurements were conducted under a nitrogen flow (50 mL/min) by using a DSC 1 (Mettler-Toledo AG, Analytical, Greifensee, Switzerland). The onset and peak temperature and enthalpy were evaluated by using STAR^®^ SW 12.10 software.

### 2.3. Preparation and Characterization of Phospholipid Complexes

#### 2.3.1. Preparation of Phospholipid Complexes

Phospholipid complexes of CW-02-79 were prepared by employing the solvent evaporation method, using soybean lecithin as the phospholipid in different mass ratios of 1:10, 1:15, and 1:20 (*w*/*w*). The required amount of CW-02-79 and lecithin was dissolved in absolute ethanol, and the obtained mixtures were refluxed for 2 h at 65 °C. After the evaporation of the solvent, using a rotary evaporator, the dried residues were gathered using a spatula and kept in Eppendorf tubes in a desiccator until characterization.

#### 2.3.2. Differential Scanning Calorimetry (DSC)

Thermal analysis was performed on pure CW-02-79, soybean lecithin, prepared phospholipid complexes, and physical mixtures containing CW-02-79 and soybean lecithin in the mass ratios of 1:10, 1:15, and 1:20. About 10 mg of the samples was measured in aluminum pans and heated from 25 to 400 °C at a rate of 10 °C/min. The measurements were conducted under a nitrogen flow (50 mL/min) by using the DSC 1 and the appropriate parameters were determined.

#### 2.3.3. Fourier Transform Infrared (FT-IR) Spectroscopy

FT-IR spectroscopy was used to investigate possible chemical interactions between CW-02-79 and soybean lecithin in the physical mixtures and obtained phospholipid complexes. For each spectrum, 10 scans were used and recorded between 4000 and 400 cm^−1^. The spectra were recorded using a Nicolet iS10 FT-IR spectrometer (Thermo Fisher Scientific, Inc., Madison, WI, USA).

#### 2.3.4. Nuclear Magnetic Resonance (NMR) Spectroscopy

This analysis was carried out on pure CW-02-79, soybean lecithin, physical mixtures, and prepared phospholipid complexes. All the compounds or mixtures were dissolved in 0.5 mL of CDCl3 and placed in an NMR tube. The proton nuclear magnetic resonance (^1^H NMR) and phosphorus nuclear magnetic resonance (^31^P NMR) spectra of the samples were acquired using a Bruker Ascend 400 spectrometer (Bruker, Berlin, Germany) at 400 MHz and 23 °C.

### 2.4. Preparation and Characterization of Intravenous Lipid Nanoemulsions

Blank and nanoemulsions loaded with CW-02-79-phospholipid complexes were prepared by using the hot high-pressure homogenization method (HPH). Briefly, an oil phase was prepared by mixing MCT and castor oil in a 1:1 (*w*/*w*) ratio, soybean lecithin, and BHT on the magnetic stirrer at 50 °C. For the CW-02-79 loaded samples, the components of the oil phase were added to the round-bottom flask containing the prepared CW-02-79-phospholipid complex and mixed until the complex was completely dissolved. An aqueous phase was prepared by dissolving polysorbate 80 as a hydrophilic stabilizer and glycerol as an isotonization agent in ultrapure water at 50 °C. Decorated nanoemulsions were prepared by adding DSPE-PEG2000 with/without DSPE-PEG2000-mannose to the oil phase. The oil phase was then added to the aqueous phase and mixed at 8000 rpm for 5 min using rotor-stator homogenizer (IKA Ultra-Turrax^®^ T25 digital, IKA^®^-Werke GmbH and Co., KG, Staufen, Germany). The coarse pre-emulsions were further processed by using a high-pressure homogenizer (EmulsiFlex-C3, Avestin Inc., Ottawa, ON, Canada) for 10 discontinuous cycles at 800 bar. The complete composition of the tested formulations is given in Table 1.

#### 2.4.1. Size Measurements and the Polydispersity Index

The droplet size (intensity-weighted mean diameter, Z-Ave) and polydispersity index (PDI) of nanoemulsions were determined using the ZetasizerNano ZS90 (Malvern Instruments Ltd., Worcestershire, UK). All measurements were carried out after adequate dilution with ultrapure water (1:500 (*v*/*v*)) at 25 °C at a fixed scattering angle of 90° using a He–Ne laser at 633 nm.

#### 2.4.2. Atomic Force Microscopy (AFM)

The morphology, shape, and size of the nanodroplets were investigated using an NTEGRA Prima Atomic Force Microscope (NT-MDT, Moscow, Russia). For the sample preparation, 10 µL of nanoemulsions diluted with ultrapure water (1:500, *v*/*v*) was placed on the circular mica substrate (Highest Grade V1 AFM Mica Discs, Ted Pella Inc., Redding, CA, USA) and dried in a vacuum dryer (2 h, 25 °C) to remove leftover water. The measurements were carried out in the intermittent contact AFM mode with NT-MDT NSGO1 silicon cantilevers (N-type, Antimony doped, Au reflective coating) using a nominal force constant of 5.1 N/m. The cantilever driving frequency was around 150 kHz throughout the testing. The topography of AFM images was analyzed using Gwyddion 2.61 (Free and Open Source software, Czech Metrology Institute, Brno, Czechia) software.

#### 2.4.3. Zeta Potential

Zeta potential (ZP) was determined using a Zetasizer Nano ZS90 by measuring droplets’ electrophoretic mobility at 25 °C and converting it into ZP by using software v7. 01. Before the measurements, the samples were diluted with ultrapure water at 1:500 (*v*/*v*) ratio.

#### 2.4.4. pH and Electrical Conductivity Measurements

Measurements of pH values were conducted at 25 °C using an HI9321 pH meter (Hanna Instruments Inc., Woonsocket, RI, USA) via direct immersion of the pH electrode into the nanoemulsions. The electrical conductivity (EC) was easily determined using the Sensio + EC 71 conductivity meter (Hach Lange GmbH, Berlin, Germany) by simple plunging of the electrode into the investigated samples.

#### 2.4.5. Osmolality

The osmolality of the samples was measured using the Advanced^®^ Model 3320 Micro-Osmometer (Advanced Instruments, Inc., Norwood, MA, USA) with a single freezing point. The nanoemulsion samples (20 µL) were collected in an Ease-Eject™ Sampler, placed at the instrument cradle, and injected into the osmometer freezing chamber.

#### 2.4.6. Encapsulation Efficacy

The encapsulation efficacy (EE) was evaluated using the Amicon Ultra-4; NMWL 10 kDa filter units (Merck Millipore, Burlington, MA, USA). Concisely, the tested nanoemulsions (2 mL) were placed in the tube and centrifuged at 2000 rcf for 45 min. The filtrate was mixed with an equal volume of isopropanol and analyzed for CW-02-79 content using the LC-MS/MS method.

#### 2.4.7. Stability Studies

To evaluate the physicochemical stability of the prepared nanoemulsions, all the formulations were stored at room temperature for three months. The visual appearance and relevant physicochemical parameters (Z-ave, PDI, ZP, pH, EC, and osmolality) were monitored using the methods described above.

### 2.5. Evaluation of the Nanoemulsion Interactions with Proteins

The interactions of the developed nanoemulsions with proteins were investigated by analyzing the changes in droplet size using the Zetasizer Nano ZS90. The nanoemulsions were incubated with bovine serum albumin (BSA, 30 mg/mL) and fetal bovine serum (FBS, 10% *v*/*v*) in phosphate buffer saline (PBS, pH 7.4) at 37 °C for 24 h. The Z-ave and PDI were measured at the beginning and after 0.5 h, 1 h, 2 h, 4 h, 8 h, and 24 h of incubation at 37 °C and compared with those of nanoemulsions incubated under the same conditions in the PBS without proteins.

### 2.6. In Vitro Release Testing of CW-02-79 from Nanoemulsions

The direct dialysis method was used for the in vitro drug release assessment. A mixture of PBS pH 7.4 and ethanol 96% *v*/*v* in the ratio of 1:1 *v*/*v* was used as a receptor medium. The addition of ethanol was necessary to address the very poor solubility of CW-02-79 in aqueous medium and to ensure sink conditions throughout the experiment. Selected optimal nanoemulsions (2 mL) were placed in a dialysis bag (cellulose membrane MW cut-off 12,000, Sigma-Aldrich Co。, St. Louis, MO, USA) and immersed in 200 mL of termostated medium (37 °C). Samples were taken at 0.5 h, 1 h, 2 h, 4 h, 8 h, and 24 h, and the concentration was determined using the LC-MS/MS technique. The release kinetics of CW-02-79 from developed nanoemulsions was evaluated by fitting the experimental data to various mathematical models (zero-order, first-order, Higuchi, Baker-Lonsdale, Korsmeyer-Peppas) using the DDSolver v. 1.0 add-in program for Microsoft Excel.

### 2.7. In Vitro Permeation Study Through the Blood–Brain Barrier

The in vitro BBB model based on the co-culture of iPSC-derived brain microvascular endothelial cells, astrocytes, and pericytes was used to estimate the transport of CW-02-79 into the brain from the developed nanoemulsions (the protocol used is available on the manufacturer’s website: iCell Blood Brain Barrier Isogenic Kit protocol, FujiFilm Cellular Dynamic Inc., Madison, WI, USA) [24]. Cell culture inserts for BBB co-cultures (pore size 0.4 µm, effective cell growth area 0.33 cm^2^, Falcon^®^, Corning Inc., Salt Lake City, UT, USA) were coated on the apical side with a fibronectin-collagen IV matrix and on the basolateral side with a 0.1% gelatin solution. Then, astrocyte and pericyte suspensions were seeded on the basolateral side, with brain microvascular endothelial cells in the apical region of inserts, followed by overnight incubation at 37 °C and 5% CO_2_. Subsequently, the iCell BMEC Plating Medium was replaced with iCell BMEC Maintenance Medium on both the apical and basolateral sides, and the medium was refreshed daily during the subsequent cultivation of the BBB. The integrity of the formed BBB was confirmed by measuring the transendothelial electrical resistance (TEER) using the EVOM2 epithelial volt/ohm meter with a chopsticks electrode (World Precision Instruments, Inc., Sarasota, FL, USA).

Selected nanoemulsions were diluted with the maintenance medium to achieve a final concentration of CW-02-79 of 3 µM in the apical compartment of the Transwell inserts and incubated at 37 °C and 5% CO_2_ for 24 h. At specific time points, after 2, 6, and 24 h, samples (100 µL) were taken from the basolateral side, and the concentration of CW-02-79 was determined by using the LC-MS/MS method. The total volume on the basolateral side was compensated with 100 µL of the fresh termostated medium. The endothelial permeability coefficient (Pe) was calculated as previously described by Heymans et al. [25]. The mass balance (%) was also determined based on the amount of CW-02-79 determined in both the apical and basolateral compartments at the end of the assay divided by the total amount of CW-02-79 placed in the apical compartment to assess the potential adsorption to plastic or non-specific binding to cells.

### 2.8. In Vivo Neuropharmacokinetic Study

A neuropharmacokinetic study was conducted in Sprague Dawley rats following intravenous administration of the selected undecorated (CW_PC_NE1) and decorated nanoemulsion (CW_PC_NE4). At the same time, the respective profiles of concentration changes in plasma were established. The study was conducted according to the National Institutes of Health Animal Care and Use Committee guidelines and approved by the Ethics Committee on Animal Experimentation of the University of Belgrade—Faculty of Pharmacy (Serbia) and Ministry of agriculture, forestry, and water management—Veterinary Directorate (323-07-10046/2020-05). Eight-week old male Sprague Dawley rats weighing 160–200 g were housed three animals per cage in the animal room, and the light/dark period was set on 12 h cycles, with the illumination 120 l×. The temperature was set at 22 ± 1 °C, with the relative humidity 40–70%. The test formulations were given intravenously in the tail vein in a volume of 5 mL/kg, obtaining a 7.5 mg/kg dose of CW-02-79. At 7 pre-determined time points (5 min, 20 min, 1 h, 3 h, 6 h, 12 h, and 24 h), 3 animals per time point were sacrificed and blood and brain samples were collected. The animals were anesthetized with ketamine hydrochloride (90 mg/kg, 10% Ketamidor, Richter Pharma AG, Wels, Austria). Blood samples were taken via cardiac puncture in heparinized syringes and spun at 1000× *g* for 10 min to separate the plasma. Simultaneously, brain was taken, weighed, and homogenized in 1 mL of methanol by using an ultrasonic probe. Homogenized samples were then centrifuged for 20 min at 3400× *g*, and the supernatants were separated. The CW-02-79 was extracted from the plasma, and the supernatants were obtained from brain via solid-phase extraction using Oasis HLB cartridges (Waters Corporation, Milford, MA, USA). Cartridges were preconditioned with methanol and water before loading the samples and internal standard. Endogenous impurities were eliminated via washing with water and methanol. The elution was performed with 1 mL of methanol for 1 min. The concentration of CW-02-79 in the eluates was determined using the LC-MS/MS technique. The concentration data were used to determine the pharmacokinetic parameters using Certara Phoenix WinNonlin™ software. v8.3.

### 2.9. LC-M/MS Method

The concentration of CW-02-79 was determined using a LC-MS/MS method. The analysis was performed on a UHPLC chromatograph ACELLA (Thermo Fisher Scientific Inc., Madison, WI, USA), coupled to a triple quadrupole mass spectrometer TSQ Quantum Access MAX (Thermo Fisher Scientific Inc., Madison, WI, USA) with a heated electrospray ionization (HESI) interface. The column was XTerra MS C18 (150 mm × 2.1 mm, 3.5 mm particle size). The mobile phase was acetonitrile/0.1% formic acid = 70:30 (*v*/*v*), the flow rate was 0.2 mL min^−1^, the column temperature was set to 35 °C, and the injection volume was 10 µL. CW-02-79 and the internal standard were detected and quantified in the positive HESI mode (*m*/*z* = 389.90–345.90 and *m*/*z* = 349.00–304.11, respectively).

### 2.10. Statistical Analysis

Where applicable, measurements were performed in triplicate, and the outcomes are presented as mean ± standard deviation (SD). After verifying the normality of the data distribution, the statistical analysis was performed using a Student’s *t*-test for two sets of results or analysis of variance (ANOVA) followed by Tukey post hoc test for more than two data sets. Statistical analysis was conducted using the IBM SPSS Statistics software (v. 25). *p*-values < 0.05 were considered statistically significant.

## 3. Results and Discussion

### 3.1. Preformulation Study

**Figure 1:**
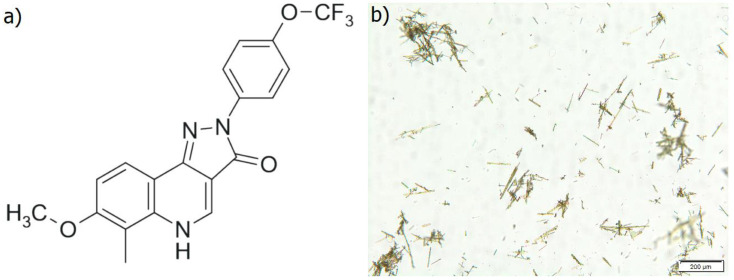
(**a**) Chemical structure of CW-02-79; (**b**) micrograph of coarse CW-02-79 powder.

CW-02-79, the novel pyrazoloquinolinone ligand with a molecular mass of 389.33 and logP value of 2.60 (Figure 1a) appeared as a yellow powder, with a broad particle size distribution (Figure 1b). Due to the limited data available in the literature, special attention was initially paid to the detailed analysis of the key physicochemical descriptors (e.g., solubility in various solvents, i.e., lipid pharmaceutical excipients and common organic solvents, melting temperature, crystallinity, and amorphization ability) in order to select an optimal strategy for the formulation development. As shown in Table S1 (Supplementary Materials), CW-02-79 is practically insoluble in water, oils, and biocompatible organic solvents, except dimethyl sulfoxide (DMSO) which can be associated with various safety concerns. In addition, since no ionizable functional groups are present, the solubility of CW-02-79 does not change with alterations of pH value. These findings exclude salt formation as an approach to improve its solubility as well as the development of conventional water- and lipid-based formulations.

To test the potential of CW-02-79 for amorphization, quenching of the melt was investigated providing an insight into the substance’s ability to become amorphous upon cooling after melting. In the first heating cycle, the thermogram of CW-02-79 exhibited two endothermic peaks at 87.95 °C and 300.84 °C (Figure S1, Supplementary Materials). The first peak represents the glass transition temperature (Tg) and indicates the possible transition from the amorphous or semi-crystalline phase to a more disordered structure. The second peak at 301.98 °C corresponds to the melting point of the crystalline structure of CW-02-79. During the subsequent rapid cooling phase, crystallization occurred with decreasing temperature, which was evident from a sharp exothermic peak at 228.46 °C. In the second heating phase, a relatively broad endothermic peak at 269.92 °C could be attributed to the melting of the crystallized form formed during the rapid cooling. These results indicate that quenching of the melt allowed crystallization to a certain extent and prevented the substance from becoming completely amorphous, excluding amorphization as the strategy for overcoming the poor water solubility.

In addition, to obtain a more detailed insight into the physical state of unprocessed CW-02-79, XRPD analysis was performed. The diffractogram of CW-02-79 displays a significant number of sharp diffraction peaks, suggesting the existence of a significant proportion of crystalline material with a well-defined lattice structure (Figure S2, Supplementary Materials). Furthermore, a broad diffraction peak extending from 3 to 30° 2θ accompanied by a raised baseline is indicative of the absence of a long-range order in the structure, suggesting the presence of amorphous material [26]. In other words, this finding could mean that the ligand under investigation is not a purely crystalline or amorphous substance, and as such, is not suitable for the development of nanocrystalline dispersions.

### 3.2. Characterization of Prepared CW-02-79—Phospholipid Complexes

Analyzing the physicochemical properties of CW-02-79, phospholipid complexation was selected as a promising approach to overcome the poor oil solubility of CW-02-79 and to develop a suitable nanoemulsion carrier. Due to its relative simplicity, the solvent evaporation technique is most commonly used for the preparation of phospholipid complexes [27]. However, there is a lack of systematic studies in the current literature that provide the optimal conditions for the preparation of phospholipid complexes. This is mainly because each substance has unique physicochemical properties, making it difficult to apply a uniform set of parameters for the formation of phospholipid complexes, such as the type of reaction solvent, phospholipids, drug to phospholipid ratio, and reaction time [28]. Due to the solubility constraints of CW-02-79, absolute ethanol was used as an organic solvent in combination with soybean lecithin as a promising phospholipid for the preparation of complexes via solvent evaporation in the different mass ratios of 1:10, 1:15, and 1:20. The obtained complexes were analyzed via DSC, FT-IR spectrometry, and NMR spectroscopy.

**Figure 2:**
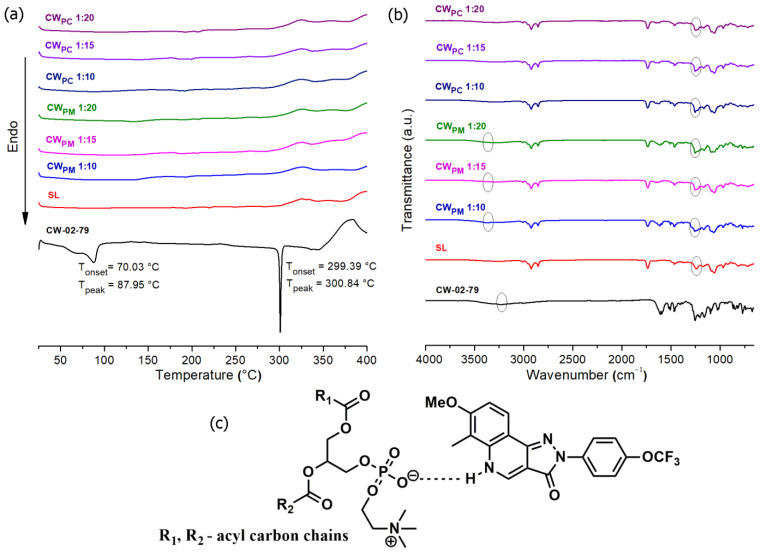
(**a**) DSC thermograms and (**b**) FT-IR spectra of pure CW-02-79, soybean lecithin (SL), physical mixtures (CW_PM_) and phospholipid complexes (CW_PC_) in investigated mass ratios of 1:10, 1:15, and 1:20; (**c**) graphical scheme of the proposed hydrogen bonding interactions between phosphatidylcholine from soybean lecithin and CW-02-79 during the CW-02-79 phospholipid complex formation.

DSC is considered a fast and reliable method to obtain information on the solid state of active substances and phospholipids and the interactions between them during the phospholipid complex formation [6]. The obtained DSC thermograms of CW-02-79, soybean lecithin, their physical mixtures, and the prepared complexes are shown in Figure 2a. No pronounced endothermic peaks were observed in the DSC curve of soybean lecithin; an exothermic peak around 325 °C could be attributed to its decomposition. On the other hand, the DSC thermograms of the physical mixtures and the CW-02-79 phospholipid complexes were highly similar and were superimposed by the lecithin curve. In other words, the characteristic sharp endothermic peak at 300.84 °C caused by the melting of CW-02-79 in its crystalline form had disappeared in both the physical mixtures and the complexes formed, probably due to masking by the soy lecithin. Therefore, additional characterization methods were required to prove the formation of the phospholipid complexes.

To identify the possible intermolecular interactions between the CW-02-79 and soybean lecithin in the physical mixtures and phospholipid complexes formed, FT-IR spectroscopy was used. The FT-IR spectrum of CW-02-79 (Figure 2b) revealed characteristic peaks at 3231 cm^−1^ (the N-H stretching vibrations), 1613 cm^−1^ (aromatic C=C stretching), 1464 cm^−1^ (C-H bending vibrations), 1254 cm^−1^ and 1092 cm^−1^ (C-O stretching vibration), and 771 cm^−1^ (out-of-plane C-H bending vibrations). The FT-IR spectrum of soybean lecithin shows characteristic peaks at 2922 cm^−1^ and 2852 cm^−1^ (C–H stretching vibration), 1737 cm^−1^ (C=O stretching), 1459 cm^−1^ (C-H bending vibrations), 1242 cm^−1^ (P=O stretching vibrations), and 1170 cm^−1^ and 1058 cm^−1^ (C-O stretching vibrations) (Figure 2b). The specific feature of CW-02-79—the N-H stretching vibration at 3231 cm^−1^—was shifted to 3376 cm^−1^ in the physical mixtures, while it almost disappeared in the phospholipid complexes due to the delocalization of the vibrations, indicating the formation of hydrogen bonds. In addition, the stretching vibration P=O bond of soybean lecithin at 1242 cm^−1^ was shifted towards 1247 cm^−1^, 1244 cm^−1^, and 1249 cm^−1^ in physical mixtures with mass ratios of 1:10, 1:15, and 1:20, respectively, while the shift was more pronounced in the corresponding phospholipid complexes (1253 cm^−1^, 1249 cm^−1^, and 1255 cm^−1^, respectively). These findings indicate that the aromatic amino group of CW-02-79 interacts with the oxygen atom of the phosphoryl group in phosphatidylcholine, the main component of soybean lecithin through hydrogen bonding in all the prepared complexes (Figure 2c).

NMR spectroscopy was used to confirm the formation of the phospholipid complex, providing an insight into the magnetic properties of hydrogen and phosphorus nuclei [6]. In the ^1^H-NMR spectra, the changes in the chemical shifts of the significant protons involved in the interactions between CW-02-79 and soybean lecithin are shown in Table 2. All the protons revealed chemical shifts, whereby the highest shift of the protons of the secondary amino group attached to the aromatic ring was observed, from 9.03 (ppm) for unprocessed CW-02-79 to 12.40 (ppm), 12.40 (ppm), and 12.57 (ppm) upon the formation of complexes at different mass ratios with soybean lecithin of 1:10, 1:15, and 1:20, respectively. Also, H-2 attached to the aromatic ring of CW-02-79 revealed certain chemical shifts upon complex formation (Table 2). To further support the ^1^H-NMR data, ^31^P-NMR was also investigated to confirm the phosphorus group’s chemical change. ^31^P-NMR revealed a phosphorus chemical shift from 0.882 to 0.993, 0.874, and 0.999 upon complex formation with different ratios of soybean lecithin (1:10, 1:15, and 1:20, respectively). All the observed downfield shifts clearly support the existence of interactions between CW-02-79 and soybean lecithin. Overall, the results of DSC, FT-IR, and NMR spectroscopy clearly confirmed the formation of complexes between CW-02-79 and soybean lecithin at all the mass ratios tested.

### 3.3. Preparation and Characterization of Nanoemulsions

The first step during the development of the nanoemulsions was to select the optimal oil core, allowing the adequate solubilization capacity for the developed phospholipid complexes as well as the stability of the nanoemulsion system per se. Among the tested oils, the highest solubility of CW-02-79 was obtained in the mixture of MCT and castor oil (1:1, *w*/*w*), and therefore, it was used as the oil phase for nanoemulsion development. The combination of MCT and castor oil, as a long-chain triglyceride, was also desirable because of the lower viscosity compared to pure castor oil as well as the free fatty acid in castor oil, which can act as co-surfactants and additional nanodroplet stabilizers [29].

Stabilizers were selected based on their suitability for parenteral administration and, more importantly, our goal to achieve the efficient delivery of CW-02-79 to the brain. Due to its biocompatibility, biodegradability, and safety reputation, soybean lecithin is the most commonly used stabilizer in commercial lipid nanoemulsions, ensuring the good stability of the system [30]. Therefore, soybean lecithin was not only used to form phospholipid complexes, but also played an important role in the stabilization of nanoemulsions. To improve the physicochemical stability during storage and thermal sterilization, polysorbate 80 was added as a co-stabilizer. In addition, numerous studies in the literature indicate that polysorbate 80 can improve the drug transport across the BBB by acting as a P-glycoprotein inhibitor, permeation enhancer, or promoter of receptor-mediated endocytosis [31]. To further facilitate the uptake of nanodroplets into the brain, a dual strategy based on passive targeting via PEGylated phospholipid (DSPE-PEG2000) and active targeting via GLUT-1 receptors using mannose at the end of the PEG chain (DSPE-PEG2000-mannose) was tested. However, due to the molecular weight and density of PEG chains, PEGylation can alter the droplet size, surface charge, and consequently the biological fate [17]. For this reason, special attention was paid to carefully selecting the optimal concentration of these PEGylated phospholipids when developing the formulation.

Immediately after preparation, all the nanoemulsions were highly fluid with a yellow appearance and characteristic bluish tint, without signs of phase separation. However, the precipitation of CW-02-79 was observed within 7 days of storage in the nanoemulsions loaded with phospholipid complexes prepared at mass ratios of 1:15 and 1:20, regardless of the stabilizer mixture used. In contrast, formulations prepared with the 1:10 CW-02-79-phospholipid complex were homogeneous with no evidence of the precipitation of CW-02-79. Therefore, the formulations prepared with 1:15 and 1:20 phospholipid complexes were excluded from further investigations.

The Z-ave and PDI of the placebo and nanoemulsions loaded with the 1:10 CW-02-79-phospholipid complex, differing in the surface composition (undecorated, decorated by DSPE-PEG2000 with/without mannose), are summarized in Table 3. The Z-ave of all the formulations tested was in the nanometer range, between 145 and 165 nm, with a PDI < 0.25, typical of a narrow size distribution, indicating good stability and suitability for intravenous administration and brain delivery (to achieve efficient brain delivery, the Z-ave of nanoparticles should be in the range of 100 to 200 nm [32]). Incorporation of the phospholipid complex into the oil core did not change the Z-ave compared to the corresponding blanks. Interestingly, no significant difference in the Z-ave and PDI was also observed between the undecorated and PEGylated formulations. Moreover, the addition of DSPE-PEG2000-mannose (CW_PC_NE4) had no effect on the Z-ave and PDI compared to the corresponding formulation prepared with DSPE-PEG2000 only (CW_PC_NE2).

**Figure 3:**
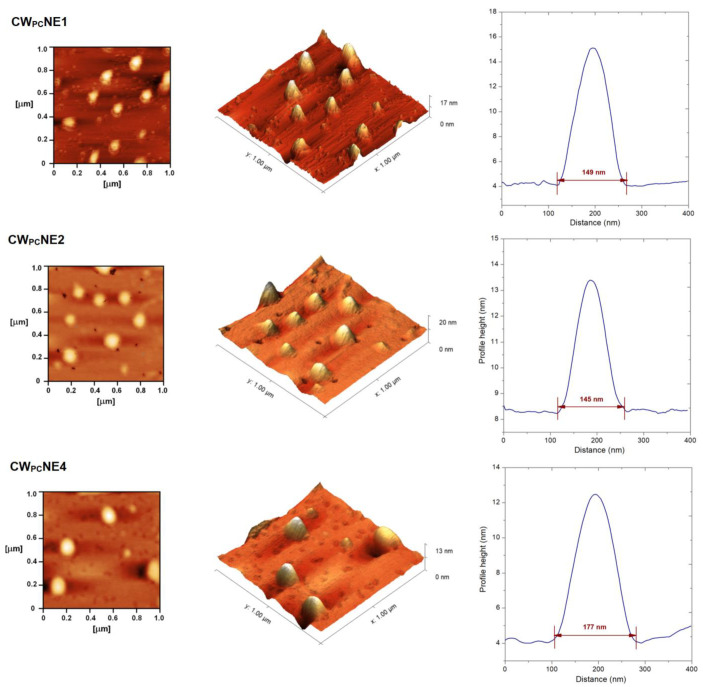
AFM images of selected nanoemulsions loaded with CW-02-79-phospholipid complex: (**a**) 2D topography of the 1.0 × 1.0 µm^2^ area of the sample, (**b**) 3D topography of 1.0 × 1.0 µm^2^ area of the sample; (**c**) height profiles of selected nanodroplets.

Although dynamic light scattering (DLS) is the most commonly used method for particle sizing, it is considered a low-resolution technique that is unable to distinguish different particle populations [16,33]. Therefore, AFM analysis was performed as a complementary method to gain further insight into the droplet size, size distribution, shape, and possible aggregation in the nanoemulsions loaded with the CW-02-79 phospholipid complex. The AFM images illustrating the 2D and 3D topography of the 1.0 × 1.0 µm^2^ area from the selected samples are presented in Figure 3a and Figure 3b, respectively. The height profiles of individual nanodroplets are shown in Figure 3c. The AFM micrographs showed droplet sizes of around 140–180 nm, with a spherical droplet morphology and a uniform shape for both PEGylated and non-PEGylated samples, which was consistent with the DLS measurements. In the formulations with 0.2% DSPE-PEG2000 as well as in the 0.1% DSPE-PEG2000-mannose combination, some sample preparation artifacts (hole-like structures) can be seen after drying, but droplets with a size of around 180 nm were still visible (Figure 3). In addition, AFM analysis proved the absence of larger droplets, aggregates, and undissolved CW-02-79 crystals in all the formulations tested.

The ZP of all the formulations developed was also measured, as absolute values of more than 30 mV indicate the good stability of the nanoemulsion system [31]. One day after preparation, the ZP values for all the tested nanoemulsions were in the range of –37.5 to –48 mV (Table 3), indicating a high negative surface charge and consequently, a strong electrostatic repulsion between the nanodroplets. In view of the fact that all the formulations were stabilized by soybean lecithin, these high values were to be expected. Interestingly, the incorporation of the CW-02-79-phospholipid complex led to a slight, but statistically significant increase in negative ZP values in all the formulations tested compared to the corresponding blank samples. In addition, the significant reduction in ZP after the addition of PEGylated phospholipids has already been described in the literature and could be attributed to the shielding effect of the PEG chains on the droplet surface.

The pH value of nanoemulsions is crucial for the physiological compatibility during administration, and particularly, for the long-term stability of the nanoemulsion system. As shown in Table 3, the pH values of all the tested nanoemulsions were suitable for parenteral administration. However, it should be noted that the pH values of the blank formulations (6.65–6.90) were higher than those of the corresponding formulations (6.14–6.33) containing the CW-02-79-phospholipid complex. This difference cannot be attributed to CW-02-79, as there are no ionizable functional groups in its structure, but to the different spatial arrangement of the lecithin molecules within the nanoemulsion system. On the other hand, the EC of the nanoemulsions loaded with CW-02-79 was similar to that of the corresponding blank samples. Finally, to avoid local damage to the vascular endothelium and circulating blood cells, the isotonicity of the nanoemulsions was adjusted by adding glycerol (1.7%, *w*/*w*). The measured osmolality of all the developed formulations was in the range of 280–300 mOsm/kg, which proves their suitability for intravenous administration.

The determination of the EE is an integral part of the physicochemical properties of the nanosystems and was performed to acquire quantitative data on the amount of encapsulated CW-02-79. The obtained results indicate that the CW-02-79-phospholipid complex was completely encapsulated in the oil phase (98.33%, 96.33%, 96.37%, and 97.89% for CW_PC_NE1, CW_PC_NE2, CW_PC_NE3, and CW_PC_NE4, respectively), with minor differences between the tested formulations. This finding clearly confirmed that the formation of phospholipid at the ratio of 1:10 significantly improved the lipophilicity of CW-02-79, retaining it in the oil core of the nanoemulsions.

### 3.4. Stability Study—Impact of Steam Sterilization and Storage

The preliminary stability study of the optimal formulations was conducted during three months of storage at 25 °C analyzing the visual appearance and the following physicochemical parameters: Z-ave, PDI, ZP, pH value, EC, and osmolality. Considering the parenteral route of administration and strict requirements for the product’s sterility, the impact of steam sterilization (autoclave, 15 min at 121 °C) was also monitored.

Interestingly, autoclaving did not significantly change the physicochemical properties of any of the developed nanoemulsions, neither immediately after preparation nor during the three-month storage period. In fact, all the nanoemulsions retained their original appearance after three months of storage, and no difference was observed between the non-autoclaved and autoclaved samples. Importantly, no CW-02-79 crystals were detected using polarization microscopy, confirming the absence of CW-02-79 precipitation. Moreover, the Z-ave and PDI remained almost unchanged during the storage period, indicating the homogeneous distribution of nanodroplets and a lack of coalescence within the system. On the other hand, immediately after autoclaving, the ZP of the undecorated formulations decreased significantly, but it changed only slightly in the PEGylated formulations. The same trend was observed over the course of three months, suggesting that PEGylated phospholipids at the droplet surface protect the negative surface charge provided by the phospholipids. However, it is important to note that the absolute ZP values of the autoclaved and non-autoclaved samples were still higher than |−30 mV| after three months of storage, demonstrating sufficient electrostatic forces for droplet repulsion.

Similarly, the pH decreased significantly after autoclaving in all the developed formulations, although the decrease was more pronounced in the undecorated formulation. This phenomenon is well described in the literature and can be attributed to the hydrolysis of phospholipids and castor oil and thus the release of fatty acids during exposure to heat [16]. The decrease in pH was accompanied by an increase in the EC, as a greater amount of free H^+^ was present in the aqueous phase. During the three-month storage period, a significant drop in pH was observed in all the formulations tested, indicating the need for variation in the selection of antioxidants. Osmolality remained in the same range suitable for intravenous administration. Overall, the results obtained indicate a satisfactory preliminary physicochemical stability of the developed nanoemulsions containing the phospholipid complex of CW-02-79. Importantly, they can withstand heat stress during steam sterilization, which is of paramount importance to ensure an adequate level of sterility, which is required for intravenous administration products.

### 3.5. Protein Binding Studies

After an intravenous injection, depending on the size, charge, and surface chemistry of the nanodroplets, proteins can bind to the their surfaces immediately after entering the bloodstream, and form a shell, the so-called protein corona. This layer can change the droplet size and their recognition via MPS, thus impairing their functionality and biological distribution. Likewise, a protein corona may trigger droplet aggregation, influencing the nanoemulsion safety [34]. To predict the behavior of the developed nanoemulsions in biological systems, we analyzed the interactions with the proteins by measuring the change in droplet size as a function of time after incubation with (i) the PBS containing BSA as the most abundant protein in plasma as well as with (ii) the PBS containing FBS, a more complex medium used in cell cultures, and rich in growth factors, hormones, amino acids, proteins, and vitamins [35].

**Figure 4:**
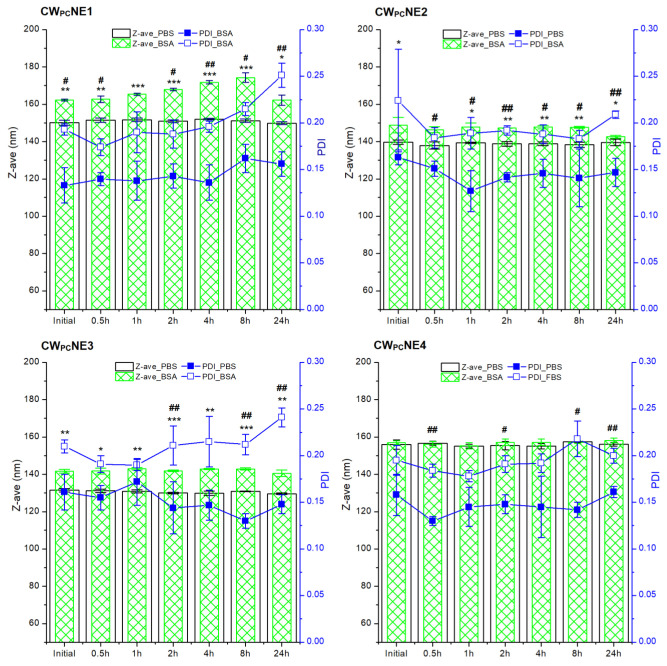
The changes in droplet size (Z-ave) and polydispersity index (PDI) of developed nanoemulsions during 24 h incubation in phosphate buffer saline (PBS)- and bovine serum albumin (BSA)-enriched PBS, reflecting the interactions of the nanodroplets with the protein-enriched media. * *p* < 0.05, ** *p* < 0.01, *** *p* < 0.001 compared to the Z-ave in PBS for each time point; # *p* < 0.05, ## *p* < 0.01, compared to the PDI values in PBS for each time point.

During the 24 h incubation of the tested nanoemulsions with the pure PBS, the Z-ave and PDI were almost constant. Therefore, the changes in these parameters after incubation with the BSA-containing medium are due to albumin binding on the surface of the nanodroplets (Figure 4). A statistically significant increase in the Z-ave and PDI was observed primarily in the undecorated CW_PC_NE1 formulation, indicating that the undecorated droplet surface has a high affinity for albumin binding. It has been shown that PEG chains can sterically hinder the access of serum proteins to the nanodroplet surface if PEGylated phospholipids are present in the optimal concentrations in the nanoformulation [18]. However, when 0.2% and 0.3% DSPE-PEG2000 (CW_PC_NE2 and CW_PC_NE3 formulations) were used, the droplet size and PDI statistically increased after incubation with BSA, indicating albumin binding to the surface. On the other hand, the interactions were less pronounced in the formulations prepared with 0.2% DSPE-PEG2000 and 0.1% DSPE-PEG2000-mannose. These results suggest that the addition of DSPE-PEG2000-mannose improves the stability of the nanodroplets in protein-enriched medium by providing additional hydrophilicity and steric hindrance.

After the incubation of the tested formulations with a medium containing FBS (10%), no significant changes were observed in the Z-ave and PDI compared to the values of these parameters measured after incubation with pure PBS (Figure S3, Supplementary Materials). Furthermore, a very slight difference was detected between the undecorated and PEGylated formulations, suggesting that FBS does not bind strongly to the surface of nanodroplets. This could be ascribed to a lower protein concentration in the FBS medium and/or competing effects between different biomolecules on the droplet surface limiting protein binding.

In summary, the results of the comprehensive characterization revealed that the dual surface decoration with DSPE-PEG2000 and DSPE-PEG2000-mannose resulted in a nanoemulsion with satisfactory physicochemical properties, stability, and low protein binding affinity (CW_PC_NE4). Therefore, it was selected as promising for evaluating the transport of CW-02-79 across the BBB in vitro and in vivo, compared to the undecorated formulation (CW_PC_NE1).

### 3.6. In Vitro Release Study

**Figure 5:**
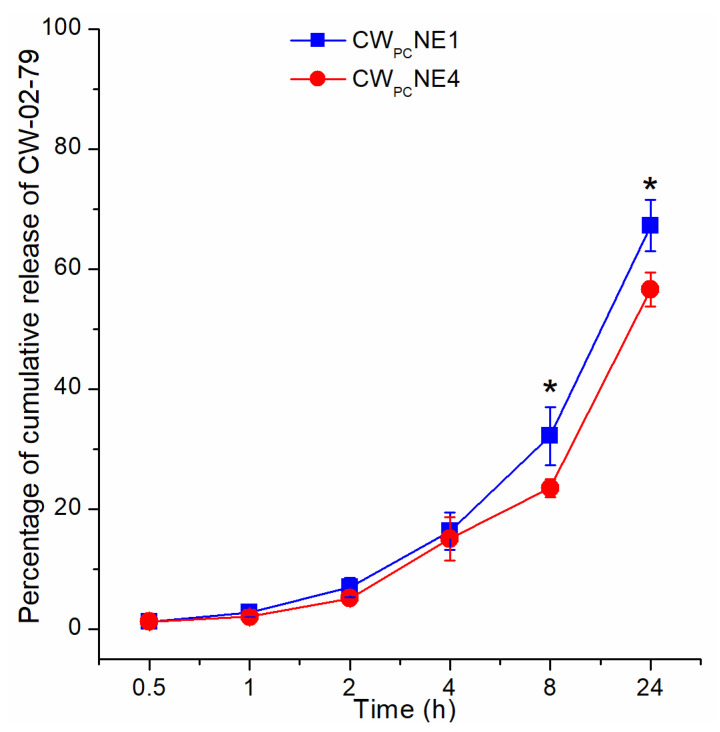
Cumulative release profile of CW-02-79 from selected nanoemulsions (CW_PC_NE1 and CW_PC_NE4). Values are shown as means ± SD (*n* = 3); * *p* < 0.05, CW_PC_NE1 vs. CW_PC_NE4.

Although not a direct indication of the drug bioavailability in vivo, the in vitro release data obtained by the direct dialysis bag method was used to evaluate how the internal structure and formulation composition of the tested nanoemulsions affect the release rate of CW-02-79, providing a deeper understanding of the complexity of the factors involved in the delivery of CW-02-79 to the brain. The obtained in vitro release profiles are shown in Figure 5. After 24 h, 67.26 ± 4.28 and 56.64 ± 2.84 (%) of CW-02-79 was released from the CW_PC_NE1 and CW_PC_NE4 formulations, respectively, indicating the sustained release of CW-02-79 from both formulations tested. It seems that CW-02-79 needs time to dissociate from the phospholipid complex, then diffuse from the oil core through the interfacial stabilizer layer and finally reach the aqueous medium [36]. However, it should be noted that the addition of PEGylated phospholipids remarkably restricted the release of CW-02-79 compared to the undecorated nanoemulsion. Since both nanoemulsions exhibited similar physicochemical properties (Z-ave, PDI, ZP, pH, and EE), the formation of a more densely packed layer at the oil–water interface could be responsible for the observed lower release rate of CW-02-79 from the CW_PC_NE4 formulation. In addition, the modeling of the release data (Table S2, Supplementary Materials) revealed that the release of CW-02-79 from both tested nanoemulsions can be best described by a first-order kinetic model. According to the literature data, this means that the release of CW-02-79 from the tested nanoemulsions follows a concentration gradient—the amount released is directly proportional to the amount remaining in the nanoemulsion and decreases with time, leading to the sustained release profile [37].

### 3.7. In Vitro Permeation Through the Blood–Brain Barrier

The BBB is responsible for the high failure rate of most new drug candidates targeting CNS disorders, as it hinders their entry into the brain in a therapeutically effective amount. Therefore, a vigorous in vitro BBB model that closely resembles the structure and functions of the human BBB is needed in the early stages of new drug/delivery system development [38]. Most BBB models declared to assess the permeability of parenteral nanoparticles are based on a monolayer of endothelial cells in the Transwell inserts [16]. However, these models do not adequately reflect the complex structure of the BBB, as barriergenic modulatory stimuli from neighboring cells are missing. Namely, a close collaboration between endothelial cells and adjacent astrocytes and pericytes is required to control the function of the tight junctions [39]. To investigate the ability of the dual-modified nanoemulsion loaded with the phospholipid complex to efficiently transport CW-02-079 across the BBB, we used an advanced in vitro iPSC-derived BBB model consisting of the co-culture of brain microvascular endothelial cells, astrocytes, and pericytes. Compared to other similar models described in the literature, the main advantages of this model are its human origin (all three cell types were differentiated from human iPSCs of the same donor), and its functional performance as assessed by the transendothelial electrical resistance (TEER) assay (>1500 Ω·cm^2^) (iCell Blood-Brain Barrier Isogenic Kit) [24]. Prior to the application of the nanoemulsions, TEER measurements were performed, and the values obtained (1616.34 ± 184.17 Ω·cm^2^) indicated satisfactory barrier integrity, according to the manufacturer’s specification. In addition, to gain insight into the status of the BBB model upon exposure to the nanoformulations, TEER values were also measured at the end of the experiment, 24 h after the application of the tested nanoemulsions. The TEER values obtained (1801.8 ± 171.23 Ω·cm^2^) did not differ remarkably, demonstrating the preserved barrier integrity and the absence of a cytotoxic effect of the tested nanoemulsions in the concentration used. 

**Figure 6:**
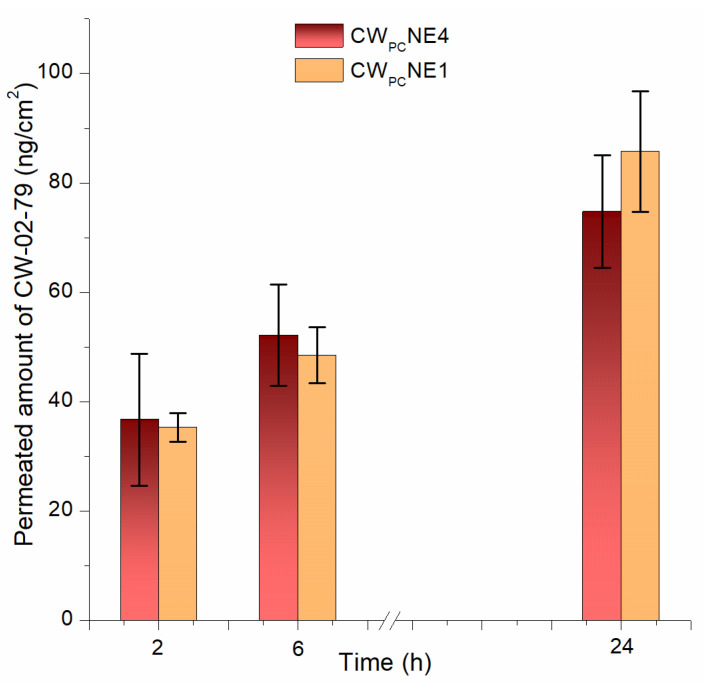
Transport of CW-02-79 through the in vitro iPSC-based blood–brain barrier model from the selected decorated (CW_PC_NE4) and undecorated (CW_PC_NE1) nanoemulsions. Values are shown as mean ± SD (*n* = 3).

The Pe of CW-02-79 from the dual functionalized nanoemulsion containing 0.2% PEG2000-DSPE and 0.1% DSPE-PEG2000-mannose (0.232 × 10^−3^ cm/min) was slightly lower compared to the plain, undecorated formulation (0.432 × 10^−3^ cm/min). No statistically significant difference was found between these two formulations in terms of the amount of CW-02-79 permeating through the BBB model used (Figure 6). These results, showing the limited permeability of CW-02-79 through the BBB, were in good agreement with the release profiles of the investigated nanoemulsions. It appears that the rate of release was the limiting factor for the efficient uptake of CW-02-79 across the BBB. Similarly, the mass balance calculations (73.58% and 78.18% for CW_PC_NE1 and CW_PC_NE4, respectively) revealed that a certain amount of CW-02-79 was retained in the endothelial cells, astrocytes, and pericytes of the BBB. Since the recovery of CW-02-79 without cells was nearly 100%, these results demonstrate the absence of non-specific binding to plastic inserts and the slower diffusion through the BBB model used. A similar result was reported for the permeation of diazepam through the BBB model consisting of bovine brain capillary endothelial cells (bBCECs) co-cultured with primary rat glial cells (astrocytes, oligodendrocytes, and microglia) [25]. However, it is evident that the expected permeation-enhancing effect of the mannose ligand, targeting the GLUT-1 receptors at the BBB, was completely absent. Therefore, the in vivo neuropharmacokinetic study provided further insights into the delivery of CW-02-79 to the brain.

### 3.8. Neuropharmacokinetic Study

To investigate the potential of the dual modification of the nanoemulsion surface with PEGylated phospholipid and the GLUT-1 targeting ligand DSPE-PEG2000-mannose to improve the delivery of CW-02-79 to the brain and its suitability for preclinical testing, a neuropharmacokinetic study was conducted in adult male rats. Based on previous studies, two favorable formulations of CW-02-79, mannose-decorated (CW_PC_NE4) and undecorated (CW_PC_NE1), were administered intravenously at a well-tolerated dose of 7.5 mg/kg. Due to the limited solubility of CW-02-79, it was not possible to prepare a solution of CW-02-79 without using a high concentration of both dimethyl sulfoxide and surfactants, so making it non-biocompatible/toxic for intravenous administration to animals.

**Figure 7:**
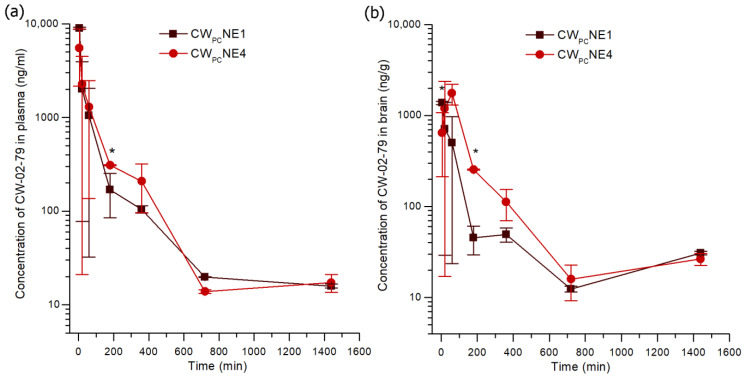
Plasma (**a**) and brain (**b**) concentration time curve after intravenous administration of selected nanoemulsions (CW_PC_NE1 and CW_PC_NE4) to male rats. Values are shown as means ± SD, *n* = 3. * *p* < 0.05 compared with CW_PC_NE1.

The mean plasma concentration curves in male rats after intravenous administration are presented in Figure 7a, and the calculated pharmacokinetic parameters were estimated via non-compartmental analysis and are summarized in Table 4. No adverse events were observed in either group after intravenous administration of the selected nanoemulsions. Blood concentrations of CW-02-79 tended to be slightly higher after administration of the decorated compared to undecorated formulation, but a statistically significant difference was found only after 3 h (*t*-test, *p* < 0.05). The area under the curve (AUC_0–24_) of CW-02-79 in plasma from CW_PC_NE4-treated rats was 1.13-fold higher than that from CW_PC_NE1 rats. On the other hand, Cmax was 1.43-fold lower in CW_PC_NE4-treated rats. These apparently discordant findings are consistent with the data on in vitro release and permeation across the BBB. The decorated nanoemulsion exhibits a slow release, as CW-02-79 must first dissociate from the phospholipid complex into oil droplets, then diffuse through the interfacial layer and finally enter the bloodstream [12]. Nevertheless, the half-life (t_1/2_) of CW-02-79 in plasma was shorter in rats treated with CW_PC_NE4 than in those treated with CW_PC_NE1. The shorter half-life of the decorated nanoemulsion could be explained by the brain delivery targeted by binding to GLUT-1 and endocytosis-mediated transport across the BBB [40], and also by the distribution of the nanodroplets in other organs and tissues.

The neuropharmacokinetic profile of CW-02-79 after the intravenous administration of a dose of 7.5 mg/kg is shown in Figure 7b. The brain concentrations were slightly higher after the administration of the decorated compared to the undecorated nanoemulsion, with the difference being significant only after 3 h (*t*-test, *p* < 0.05), which follows the pattern seen in plasma. The brain-to-plasma partition coefficients for the decorated and undecorated nanoemulsion were 43.6% and 33.1%, respectively, and reflect moderate levels of brain exposure [41]. Introducing dual surface decoration with PEGylated phospholipids and DSPE-PEG2000-mannose in the nanoemulsion preparation resulted in an increase in brain AUC_0–24_ values of 48.5%; however, the difference was statistically insignificant.

When interpreting the presented partial proof of concept that the surface decoration of the nanoemulsion with the GLUT-1-targeting ligand DSPE-PEG2000-mannose can enhance the GLUT-1-mediated permeation through the BBB, two caveats should be noted. First, for a complete insight into the brain exposure, the ratio of unbound drug in the brain to unbound drug in the plasma would need to be determined [42]. Second, a recent study by Lei et al. [21] showed that the glycemic control strategy plays an important role in shifting GLUT-1 from the apical to the basolateral side of the BBB, allowing the mannose-decorated nanodroplets to penetrate the BBB and accumulate in the brain. In addition, the complex and sex-dependent role of σ2 receptors in metabolic regulation was recently discovered in mice [43]. In our study, the blood glucose level of rats was not controlled, which might be related to the suboptimal permeation of mannose-decorated nanoemulsions into the brain. Therefore, further studies should aim to evaluate the unbound brain-to-plasma partition coefficients of the discovered σ2 receptor ligand in the settings of controlled glycemia in rats of both sexes and possibly with different concentrations of DSPE-PEG2000-mannose.

## 4. Conclusions

Due to its limited water and oil solubility, it was not possible to prepare a conventional delivery vehicle for the novel pyrazoloquinolinone ligand CW-02-79 without using non-biocompatible solvents. Based on systemic exploration, a two-fold formulation strategy was applied, combining phospholipid complexes and lipid nanoemulsions to improve the liposolubility of CW-02-79 by introducing decoration to enhance the brain-targeting efficiency. The developed biocompatible nanoemulsion loaded with the CW-02-079—phospholipid complex at a mass ratio of 1:10 exhibited a small droplet size and narrow size distribution as well as satisfactory physicochemical stability during steam sterilization and short-term storage at 25 °C, making it suitable for intravenous administration. The analysis of protein-binding interactions revealed that the PEGylated nanoemulsions had fewer observable interactions compared to the undecorated nanoemulsions, especially when 0.2% DSPE-PEG2000 and 0.1% DSPE-PEG2000-mannose were combined. The neuropharmacokinetic study showed that there were minor differences in the pharmacokinetic parameters favoring the dual surface-functionalized nanoemulsion with PEGylated phospholipids and the GLUT-1-targeting ligand DSPE-PEG2000-mannose to facilitate BBB crossing and enhance transport into the brain. The study suggests, overall, that this formulation is suitable for the preclinical testing of ligands such as CW-02-79, but further research is required to optimize their targeted delivery to the brain.

## Figures and Tables

**Table 1:** Composition of optimal placebo and nanoemulsions loaded with CW-02-79—phospholipid complex.

Composition (%, *w*/*w*)	NE1	CW_PC_NE1	NE2	CW_PC_NE2	NE3	CW_PC_NE3	CW_PC_NE4
Oil phase							
CW_PC_	-	1.65	-	1.65	-	1.65	1.65
MCT	10	10	10	10	10	10	10
Castor oil	10	10	10	10	10	10	10
Soybean lecithin	0.5	0.5	0.5	0.5	0.5	0.5	0.5
DSPE-PEG2000	-	-	0.2	0.2	0.3	0.3	0.2
DSPE-PEG2000-mannose	-	-	-	-	-	-	0.1
BHT	0.05	0.05	0.05	0.05	0.05	0.05	0.05
Aqueous phase							
Polysorbate 80	2	2	2	2	2	2	2
Glycerol	1.7	1.7	1.7	1.7	1.7	1.7	1.7
Ultrapure water ad	100	100	100	100	100	100	100

CW_PC_: CW-02-79-phospholipid complex in a mass ratio of 1:10 (*w*/*w*); MCT: medium-chain triglycerides; BHT: butylhydroxytoluene.

**Table 2:** Overview of the chemical shifts (in ppm) of pure CW-02-79 or soybean lecithin compared to prepared phospholipid complexes (CW_PC_ 1:10, 1:15, and 1:20) using ^1^H-NMR and ^31^P-NMR.

	CW-02-79/SL *	CW_PC_ 1:10	CW_PC_ 1:15	CW_PC_ 1:20
^1^H-NMR				
NH	9.03, bs	12.40, bs	12.40, bs	12.57, bs
H-2	8.37 (d, J = 6.3 Hz),	8.63, s	8.69, s	8.66, s
H-5	8.28–8.23, m	8.07 (d, J = 8.8 Hz)	8.14 (d, J = 8.8 Hz)	8.11 (d, J = 8.8 Hz)
H-6	7.14 (d, J = 8.8 Hz),	6.98 (d, J = 9.0 Hz)	7.04 (d, J = 8.9 Hz)	7.01 (d, J = 9.1 Hz)
CH3	2.40, s	2.40, s	2.45, s	2.44, s
OCH3	3.97, s	3.85, s	3.90, s	3.88, s
H-2′ and H-6′	8.28–8.23, m	8.21 (d, J = 9.0 Hz)	8.25 (d, J = 9.0 Hz)	8.25 (d, J = 8.9 Hz)
H-3′ and H-5′	7.29 (d, J = 8.7 Hz)	7.23 (d, J = 8.6 Hz)	* covered by CDCl3 signal	7.24 (d, J = 8.6 Hz)
^31^P-NMR				
P=O	0.882	0.993	0.874	0.999

* ^1^H-NMR refers to CW-02-79, while ^31^P-NMR refers to soybean lecithin.

**Table 3:** Droplet size (Z-ave), polydispersity index (PDI), zeta potential (ZP), pH, and electrical conductivity (EC) of autoclaved and non-autoclaved blank and nanoemulsions loaded with CW-02-79–phospholipid complex after preparation and after three months of storage at 25 °C.

Sample		Z-ave (nm)	PDI	ZP (mV)	pH	EC (μS/cm)
One day after preparation						
NE1	n	152.8 ± 1.3	0.148 ± 0.016	−45.1 ± 0.2	6.70 ± 0.02	81.4 ± 0.2
	a	152.6 ± 0.5	0.148 ± 0.016	−37.8 ± 1.1 ^e^	5.54 ± 0.01 ^f^	82.5 ± 0.6 ^e^
CW_PC_NE1	n	151.3 ± 1.3	0.174 ± 0.035	−42.3 ± 1.2 ^a^	6.19 ± 0.01 ^c^	79.2 ± 1.0 ^b^
	a	148.0 ± 1.0	0.143 ± 0.010	−32.6 ± 1.3	5.56 ± 0.01	72.3 ± 0.2
NE2	n	145.5 ± 1.8	0.112 ± 0.019	−46.0 ± 0.4	6.90 ± 0.01	82.4 ± 0.3
	a	144.1 ± 0.2	0.104 ± 0.018	−43.1 ± 1.4	5.53 ± 0.01	90.6 ± 0.4
CW_PC_NE2	n	158.4 ± 3.4 ^b^	0.217 ± 0.007 ^b^	−48.0 ± 0.4 ^c^	6.33 ± 0.02	90.9 ± 0.2 ^c^
	a	155.0 ± 0.1	0.140 ± 0.005 ^f^	−40.9 ± 1.2 ^e^	5.54 ± 0.02 ^e^	94.9 ± 0.1 ^f^
NE3	n	161.2 ± 2.0	0.158 ± 0.025	−37.5 ± 0.4	6.65 ± 0.03	96.9 ± 0.3
	a	159.7 ± 1.4	0.133 ± 0.011	−39.2 ± 0.2	5.76 ± 0.01	102.1 ± 0.4
CW_PC_NE3	n	144.7 ± 2.5 ^b^	0.173 ± 0.025	−42.0 ± 0.5 ^c^	6.14 ± 0.02 ^c^	96.6 ± 0.3 ^c^
	a	139.3 ± 2.1	0.119 ± 0.034	−42.7 ± 0.7	5.85 ± 0.02 ^e^	100.4 ± 0.3 ^e^
CW_PC_NE4	n	153.4 ± 0.9	0.113 ± 0.090	−37.9 ± 0.3	6.31 ± 0.01	101.7 ± 0.2
	a	155.2 ± 1.7	0.129 ± 0.024	−40.1 ± 0.3	5.90 ± 0.03 ^e^	113.3 ± 0.1 ^f^
After three months of storage						
NE1	n	150.0 ± 2.3	0.139 ± 0.014	−31.5 ± 0.7	5.92 ± 0.04	126.6 ± 0.9
	a	146.5 ± 1.7	0.135 ± 0.008	−31.7 ± 0.5	5.17 ± 0.01	89.0 ± 0.9
CW_PC_NE1	n	146.7 ± 1.4	0.108 ± 0.022	−40.6 ± 1.9	4.75 ± 0.04 ^i^	135.4 ± 1.9 ^i^
	a	142.4 ± 1.0	0.122 ± 0.021	−30.9 ± 0.6	4.97 ± 0.04	84.7 ± 1.5
NE2	n	142.7 ± 1.8	0.111 ± 0.008	−30.3 ± 0.3	4.68 ± 0.01	115.4 ± 0.3
	a	145.6 ± 0.6	0.092 ± 0.006	−43.1 ± 1.1	5.17 ± 0.00	112.1 ± 0.3
CW_PC_NE2	n	154.8 ± 0.8	0.146 ± 0.01 ^h^	−40.3 ± 0.3 ^i^	5.73 ± 0.01 ^i^	123.5 ± 0.6 ^i^
	a	153.2 ± 1.0	0.135 ± 0.026	−31.2 ± 1.9	5.22 ± 0.01	114.0 ± 0.7
NE3	n	165.5 ± 1.2	0.129 ± 0.026	−40.0 ± 0.2	5.12 ± 0.02	179.9 ± 0.1
	a	164.6 ± 1.1	0.159 ± 0.008	−46.0 ± 0.3	5.14 ± 0.01	120.9 ± 0.5
CW_PC_NE3	n	148.5 ± 1.8	0.166 ± 0.009	−49.7 ± 1.9 ^h^	5.17 ± 0.03 ^i^	147.8 ± 1.1 ^i^
	a	139.6 ± 1.0	0.147 ± 0.023	−36.3 ± 1.2	5.25 ± 0.01	113.0 ± 0.7
CW_PC_NE4	n	158.0 ± 1.8 ^g^	0.152 ± 0.017 ^g^	−40.6 ± 0.5 ^i^	5.13 ± 0.01 ^h^	171.1 ± 0.9 ^h^
	a	155.0 ± 2.0	0.137 ± 0.035	−39.9 ± 0.3	4.94 ± 0.02	107.9 ± 1.1

n—non-autoclaved nanoemulsion sample, a—autoclaved nanoemulsion sample. ^a^ *p* < 0.05, ^b^ *p* < 0.01, ^c^ *p* < 0.001 compared to corresponding blank sample. ^d^ *p* < 0.05, ^e^ *p* < 0.01, ^f^ *p* < 0.001 compared with corresponding non-autoclaved sample. ^g^ *p* < 0.05, ^h^ *p* < 0.01, ^i^ *p* < 0.001 compared with the same sample one day after preparation.

**Table 4:** Calculated pharmacokinetic parameters of CW-02-79 after intravenous administration of selected nanoemulsions CW_PC_NE1 and CW_PC_NE4. Values are shown as means ± SD, *n* = 3.

Parameter	Formulation	
	CW_PC_ NE1	CW_PC_ NE4
Plasma		
C_max_ (ng/mL)	8964.35 ± 250.78	6280.30 ± 2265.22
T_max_ (h)	0.083 ± 0.00	0.16 ± 0.14
AUC_0–24_ (h·ng/mL)	5934.46 ± 1082.25	6680.29 ± 1396.42
t_1/2_ (h)	6.48 ± 1.35	3.53 ± 0.63 *
Brain		
C_max_ (ng/g)	1377.53 ± 58.32	1279.49 ± 1069.41
T_max_ (h)	0.083 ± 0.0	1.22 ± 1.53 *
AUC_0–24_ (h·ng/g)	1961.74 ± 712.00	2913.77 ± 985.67
t_1/2_ (h)	6.37 ± 3.13	4.78 ± 0.93

C_max_: maximum concentration; T_max_: maximum concentration; t_1/2_: elimination half-life; AUC_0–t_: area under the curve from time zero to 24 h. * *p* < 0.05 compared with CW_PC_NE1.

## Data Availability

The original contributions presented in this study are included in the article/Supplementary Materials. Further inquiries can be directed to the corresponding author(s).

## Supplementary Materials

The following supporting information can be downloaded at: https://www.mdpi.com/article/10.3390/pharmaceutics17020232/s1. Table S1. Solubility of CW-02-79 in the selected solvents, Table S2. Interpretation the release kinetics of selected nanoemulsions by fitting in vitro drug release data to different models, Figure S1. XRPD pattern of unprocessed CW-02-79, Figure S2. DSC thermograms showing the crystallization tendency of CW-02-79 after melt quenching, Figure S3. The changes of droplet size (Z-ave) and polydispersity index (PDI) of developed nanoemulsions during 24-h incubation in phosphate buffer saline (PBS) and fetal bovine serum (FBS) enriched PBS, reflecting the interactions of the nanodroplets with the biological media.

## Abbreviations

The following abbreviations are used in this manuscript:

AFM	Atomic Force Microscopy
BBB	Blood Brain Barrier
bBCEC	Bovine Brain Capillary Endothelial Cell
BHT	Butylhydroxyltoluene
BMEC	Brain Microvascular Endothelial cells
BSA	Bovine Serum Albumin
CW_PC_	CW-02-79—phospholipid complex
CW_PM_	CW-02-79—physical mixture
DLS	Dynamic Light Scattering
DMSO	Dimethyl Sulfoxide
DSC	Differential Scanning Calorimetry
EC	Electrical Conductivity
EE	Encapsulation Efficiency
FBS	Fetal Bovine Serum
FT-IR	Fourier Transform Infrared Spectroscopy
GABA_A_	γ-aminobutyric acid type A
GLUT-1	Glucose Transporter-1
HLB	Hydrophilic–Lipophilic Balance
HPH	High-Pressure Homogenization
iPSCs	Induced Pluripotent Stem Cells
LC-MS/MS	Liquid Chromatography with Tandem Mass Spectrometry
MCT	Medium-Chain Triglycerides
MPS	Mononuclear Phagocytic System
MW	Molecular Weight
NMR	Nuclear Magnetic Resonance
PBS	Phosphate Buffer Saline
PDI	Polydispersity Index
PDSP	Psychoactive Drug Screening Program
Pe	Permeability Coefficient
ppm	Parts Per Million
SD	Standard Deviation
TEER	Trans Endothelial Electrical Resistance
UHPLC	Ultra-High-Performance Liquid Chromatography
XRPD	X-Ray Powder Diffraction
Z-ave	Intensity-Weighted Mean Hydrodynamic diameter
ZP	Zeta Potential
σ2	Sigma-2 Receptors
