# Supplementary material for: Intravenous Nanoemulsions Loaded with Phospholipid Complex of a Novel Pyrazoloquinolinone Ligand for Enhanced Brain Delivery
Source: Pharmaceutics. 2025 Feb 11;17(2):232. doi: 10.3390/pharmaceutics17020232 (PMC11858841; doi:10.3390/pharmaceutics17020232)
Supplement: Supplementary file 1 [file pharmaceutics-17-00232-s001.zip › pharmaceutics-3429538-supplementary.pdf]

Supplementary material

Intravenous nanoemulsions loaded with phospholipid complex of a novel pyrazoloquinolinone ligand for enhanced brain delivery

Tijana Stanković[1], Tanja Ilić[1,*], Branka Divović Matović[2], Milos Petkovic[3], Vladimir Dobričić[4], Ivan Jančić[5], Biljana Bufan[5], Kristina Jezdić[2], Jelena Đoković[1], Ivana Pantelić[1], Danijela Randjelović[6], Dishary Sharmin[7], James M. Cook[7], Miroslav M. Savić[2], Snežana Savić[1]

[1] Department of Pharmaceutical Technology and Cosmetology, Faculty of Pharmacy, University of Belgrade, 11211 Belgrade, Serbia

[2] Department of Pharmacology, Faculty of Pharmacy, University of Belgrade, 11211 Belgrade, Serbia

[3] Department of Organic Chemistry, Faculty of Pharmacy, University of Belgrade, 11211 Belgrade, Serbia

[4] Department of Pharmaceutical Chemistry, Faculty of Pharmacy, University of Belgrade, 11211 Belgrade, Serbia

[5] Department of Microbiology and Immunology, Faculty of Pharmacy, University of Belgrade, 11211 Belgrade, Serbia

[6] Institute of Chemistry, Technology and Metallurgy, National Institute of the Republic of Serbia, University of Belgrade, 11000 Belgrade, Serbia

[7] Department of Chemistry and Biochemistry, Milwaukee Institute for Drug Discovery, University of

Wisconsin-Milwaukee, Milwaukee, WI 53211, USA

* Correspondence: tanja.ilic@pharmacy.bg.ac.rs; Tel.: +381-11-3951-365

Table S1. Solubility of CW-02-79 in the selected solvents (mean ± SD, *n* = 3).

Solvent	Solubility (μg/ml)
MCT	49.93±0.07
Castor oil	27.96±0.03
MCT:castor oil, (1:1, *w*/*w*)	218.38±0.30
Soybean oil	65.68±0.43
MCT:soybean oil (1:1, *w*/*w*)	41.41±0.04
Fish oil	34.19±0.06
Methanol	939.09±3.53
Isopropanol	5,746.55±45.8
DMSO	>30000
0.1M HCl	12.33±0.61
Phosphate buffer, pH 7.4	11.80±0.70
Ultrapure water	11.75±0.20

Table S2. Interpretation the release kinetics of selected nanoemulsions by fitting in vitro drug release data to different models

Kinetic model	Parameter	Formulation	
		**CW$_{PC}$NE1**	**CW$_{PC}$NE4**
Zero-order model	K	0.049	0.041
	R^2	0.9665	0.9784
	$R^2_{adjusted}$	0.9665	0.9784
	AIC	30.06	25.23
First-order model	**K**	**0.001**	**0.001**
	**R^2**	**0.9970**	**0.9958**
	**$R^2_{adjusted}$**	**0.9970**	**0.9958**
	**AIC**	**15.54**	**15.39**
Higuchi model	K	1.525	1.256
	R^2	0.8845	0.8745
	$R^2_{adjusted}$	0.8845	0.8745
	AIC	37.49	35.80
Korsmeyer Peppas model	K	0.202	0.133
	n	0.801	0.833
	R^2	0.9902	0.9971
	$R^2_{adjusted}$	0.9877	0.9935
	AIC	24.69	20.08
Baker Lonsdale model	K	0.000	0.000
	R^2	0.8430	0.8409
	$R^2_{adjusted}$	0.8430	0.8409
	AIC	39.33	37.22

K – release rate constant, R^2 –coefficient of determination, $R^2_{adjusted}$ - adjusted coefficient of determination, AIC – Akaike Information Criterion, n – diffusion release exponent

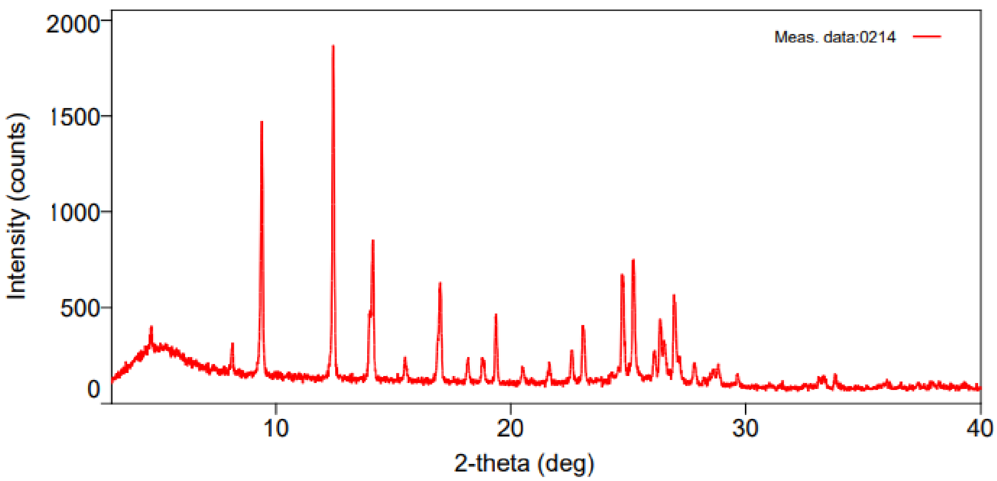

Figure S1. XRPD pattern of unprocessed CW-02-79

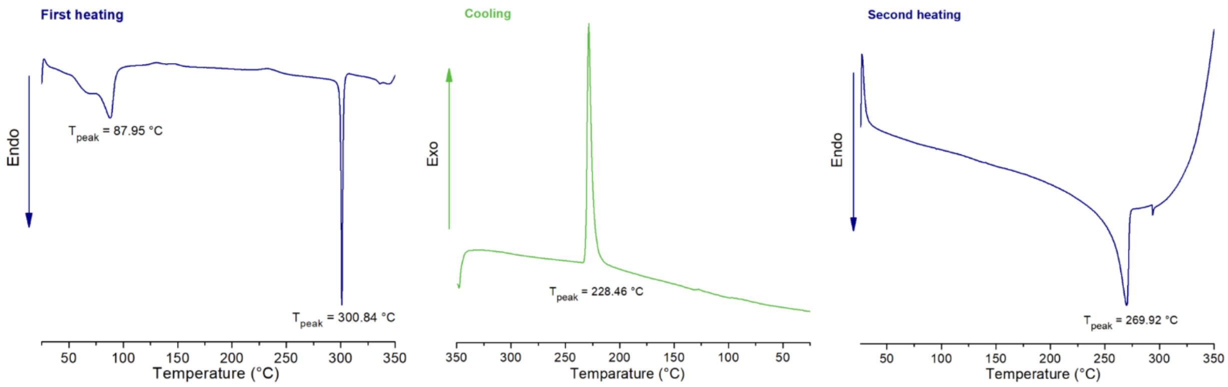

Figure S2. DSC thermograms showing the crystallization tendency of CW-02-79 after melt quenching

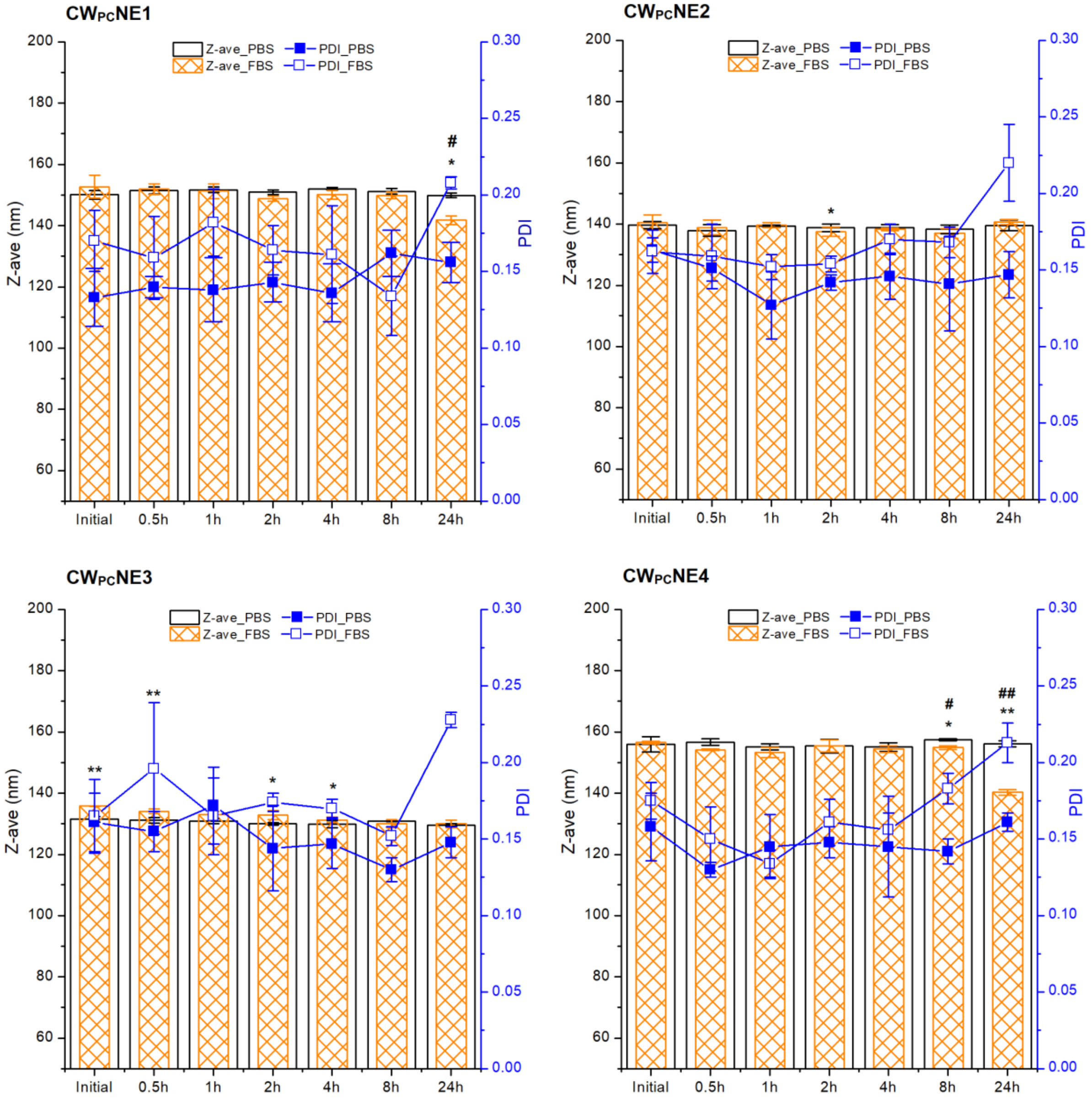

Figure S3. The changes of droplet size (Z-ave) and polydispersity index (PDI) of developed nanoemulsions during 24-hour incubation in phosphate buffer saline (PBS) and fetal bovine serum (FBS) enriched PBS, reflecting the interactions of the nanodroplets with the biological media. *$p < 0.05$, ** $p < 0.01$ compared to the Z-ave in PBS for each time point; # $p < 0.05$, ## $p < 0.01$ compared to the PDI values in PBS for each time point
